# The effect of time interval between antenatal corticosteroid administration and delivery on outcomes in late preterm neonates born to mothers with diabetes: a retrospective cohort study

**DOI:** 10.3389/fped.2023.1239977

**Published:** 2023-08-25

**Authors:** Xiaoyu Li, Jing Zhang, Qingfei Hao, Yanna Du, Xiuyong Cheng

**Affiliations:** Department of Neonatology, The First Affiliated Hospital of Zheng Zhou University, Zhengzhou, China

**Keywords:** antenatal corticosteroids, gestational diabetes mellitus, late preterm, hypoglycemia, respiratory system diseases

## Abstract

**Objectives:**

The study aims to investigate whether the time interval between administering antenatal corticosteroids (ACS) and delivery influences the neonatal outcomes in late preterm (LPT) neonates (34 + 0 to 36 + 6 weeks) born to mothers with diabetes.

**Study design:**

This retrospective cohort study included women with any type of diabetes who gave birth between 34 + 0 weeks and 36 + 6 weeks of gestation. Based on the time interval between the first dose of corticosteroid and delivery, the cases were stratified into the following groups: <2, 2–7, and >7 days. Women unexposed to ACS served as the control group. The primary outcomes included the incidence of neonatal hypoglycemia and respiratory distress syndrome/transient tachypnea of the newborn. Multivariate logistic regression was used to assess the relationship between the time interval and neonatal outcomes and adjust for potential confounders.

**Results:**

The study enrolled a total of 636 parturients. Among them, 247 (38.8%) delivered within 2 days after ACS administration, 169 (26.6%) within 2–7 days, and 126 (19.8%) at >7 days. Baseline characteristics such as type of diabetes, methods of glycemic control, preterm premature rupture of membrane, placenta previa, cesarean delivery, indication for delivery, percentage of large for gestational age, birth weight, and HbA1c in the second or third trimester were significantly different among the four groups. The multivariate analysis showed no statistically significant difference in the incidence of primary or secondary neonatal outcomes between the case and control groups.

**Conclusions:**

ACS treatment was not associated with neonatal hypoglycemia and respiratory outcomes in LPT neonates born to diabetic mothers, regardless of the time interval to delivery.

## Introduction

In the country, late preterm (LPT: 34 + 0 to 36 + 6 weeks gestation) births comprise approximately 70% of all preterm births (PTB) (1). In addition, this subgroup of infants has a higher risk of suffering from multiple complications than those born at term, such as respiratory distress, leading to respiratory distress syndrome (RDS) and transient tachypnea of the newborn (TTN) (2, 3). In recent years, the prevalence of diabetes mellitus and gestational diabetes mellitus (GDM) has gradually increased in mainland China, and GDM has become a common complication during pregnancy (4). Maternal diabetes [including GDM or diabetes in pregnancy (DIP)] is associated with delayed surfactant synthesis and increased rates of cesarean delivery, which can also lead to increased rates of neonatal RDS and TTN (5–7).

The Antenatal Late Preterm Steroid (ALPS) trial, a large multicenter randomized controlled trial, found that using antenatal corticosteroids (ACS) was significantly associated with reduced complications in the respiratory system of LPT neonates (8). Other studies also confirmed this finding (9–13). Accordingly, many physicians have started routinely using ACS for women at risk of LPT labor (14). However, these studies assessing the efficacy of ACS largely excluded women with diabetes (including GDM and DIP) (10, 11) or included only a small proportion of them (8, 9, 15). ACS may be important in improving respiratory outcomes in infants born to mothers with diabetes. Furthermore, ACS was found to be associated with an increased risk of neonatal hypoglycemia. Hypoglycemia is also a common complication in newborns born to diabetic mothers (7, 16, 17). Therefore, it is necessary to evaluate the efficacy of ACS in women with diabetes who delivered during the LPT period.

Recently, several retrospective studies on the safety and efficacy of ACS treatment in women with diabetes (GDM or DIP) who delivered during the LPT period have obtained inconsistent results (18–20). More importantly, none of those studies specifically assessed the effect of the time interval between corticosteroid administration and delivery on neonatal outcomes, which plays an important role in the clinical effectiveness of ACS. Thus, our study aims to determine if the time interval between ACS administration and delivery influences the outcomes of LPT neonates born to diabetic mothers.

## Materials and methods

### Data collection

This retrospective cohort included women diagnosed with GDM and DIP (including pre-existing DIP) and who delivered at 34 to 36 + 6 weeks of pregnancy in a tertiary medical center between 1 October 2017 and 31 December 2022. The exclusion criteria included women with multiple pregnancies, fetal chromosomal abnormalities, or major fetal anomalies. According to the time interval between the first dose of ACS and delivery, the cases were divided into the following groups: <2, 2–7, and >7 days. Women unexposed to prenatal steroids served as the control group. The demographic and obstetric variables recorded include the type of diabetes, method of glycemic control, maternal age, gravidity, parity, preterm premature rupture of membrane (PPROM), placenta previa, mode of delivery, indication for delivery, body mass index (BMI), hypertensive disorders, second- or third-trimester glycated hemoglobin (HbA1c), and indication for ACS. Neonatal baseline data included male sex, gestational age (GA) at ACS, GA at delivery, birth weight, small or large for gestational age (LGA), and 1 min Apgar score. The primary outcomes included the incidence of RDS/TTN and neonatal hypoglycemia. The secondary outcomes included the lowest glucose level, neonatal ward admission, admission due to respiratory issue, admission due to hypoglycemia, duration of hospitalization, oxygen requirement (any form of oxygen requirement after birth), continuous positive airway pressure (CPAP) or high flow nasal cannula (HFNC), mechanical ventilation, requirement of resuscitation at birth, and need for surfactant.

### Definitions

The diagnosis of GDM or DIP was based on one or more of the following abnormal glucose values: GDM: fasting blood glucose ≥5.1 mmol/L, 1-h post 75 g oral glucose tolerance test (OGTT) ≥ 10.0 mmol/L, and 2-h post 75 g OGTT ≥ 8.5 mmol/L; DIP: fasting glucose ≥7 mmol/L and 2-h glucose ≥11.1 mmol/L, according to the World Health Organization criteria (21). The Fenton 2013 curve was used to calculate the birth weight percentile (22). RDS was characterized as follows: (1) progressive dyspnea accompanied by an expiratory groan, cyanosis, or inspiratory three-concave sign within 6 h after birth and (2) reduced transparency of both lungs, an air bronchogram sign, indistinct septal margins, an indistinct heart, or white lungs on chest x-rays (23). The main chest x-ray findings of TTN were interstitial, alveolar, and interlobular pleural effusion. Hypoglycemia was defined as glucose levels below 2.2 mmol/L within 24 h after birth (24). All newborns with a glucose level ≤2.6 mmol/L subsequently received a protocol including intravenous dextrose therapy and monitoring in our unit. For newborns without hypoglycemic symptoms, blood glucose monitoring should be conducted after the initial feeding (within 1.5 h after birth), and pre-feeding blood glucose should be checked every 3–6 h within 24 h after birth. Newborns with hypoglycemic symptoms require continuous blood glucose monitoring.

ACS exposure was defined as at least one dose of dexamethasone (6 mg) given at any time during pregnancy. A complete course was defined as four intramuscular 6-mg doses of dexamethasone administered 12 h apart. Indications for ACS (25) included preterm labor (regular uterine contractions leading to cervical changes), PPROM, fetal indications (such as placental insufficiency, intrauterine growth restriction, or oligohydramnios), maternal indications (such as GDM or DIP and hypertensive disorders), abnormal vaginal bleeding (such as placenta previa and placental abruption), and asymptomatic changes in the cervix (cervical dilation of >4 cm and/or length of <15 mm). However, prophylactic treatment with dexamethasone was ultimately the decision of the obstetrician.

### Statistical analysis

Data analysis was performed using SPSS version 26.0 software. ANOVA test and chi-squared tests were used to compare continuous and categorical variables, respectively. *Post hoc* analysis was performed using the Bonferroni test to correct for multiple comparisons. The least significant difference test or Tamhane's test was used to compare any two groups according to homogeneity of variance or heterogeneity of variance, respectively. A two-tailed test was used to evaluate the significance of statistical tests at the significance level of 5%. Multivariate logistic regression analysis was performed to assess the relationship between the interval from ACS administration to birth and the neonatal outcomes and to adjust for potential confounders. In the multivariate analysis, the regression model included variables with differences between groups (*P* < 0.05).

## Results

Overall, the study enrolled a total of 636 parturients. Among them, 247 (38.8%) delivered within 2 days after ACS administration, 169 (26.6%) within 2–7 days, and 126 (19.8%) at >7 days. Table 1 shows the baseline characteristics of each group. Baseline characteristics such as type of diabetes, methods of glycemic control, PPROM, placenta previa, cesarean delivery, indication for delivery, percentage of LGA, birth weight, and HbA1c in the second or third trimester were significantly different among the four groups (Table 1). The control group and the <2 days group were more likely to deliver due to spontaneous indications, while the other two groups primarily due to maternal indications. Compared with the other three groups, neonates born after 7 days of ACS treatment had a lower birth weight (2,548.3 ± 509.6, >7 days group, vs. 2,892.5 ± 677.1, control group; 2,806.6 ± 564.5, <2 days group; 2,754.5 ± 582.1, 2–7 days group; *P* < 0.001). There were no other significant differences in baseline characteristics among the four groups. Women were more likely to receive ACS due to maternal indications (Table 2). Table 3 shows the neonatal outcomes. In the unadjusted estimates, there were no differences in the rates of primary and secondary neonatal outcomes among the four groups, except surfactant use and neonatal ward admission. *Post hoc* analysis showed that there was a statistical difference in the rate of surfactant use between the <2 days group and the >7 days group (2.0% vs. 8.7%, *P* = 0.026), but no statistical difference in the hospitalization rate was found between any two groups. We used multivariate logistic regression analysis to control for different baseline characteristics among groups and to evaluate the association between the interval from ACS administration to birth and the neonatal outcomes. Table 4 presents the results. The multivariate analysis revealed no statistically significant difference in the incidence of primary or secondary neonatal outcomes between the case and control groups.

**Table 1:** Characteristics of the study population.

Variables	No antenatal corticosteroids	Antenatal corticosteroids			*P*
		Less than 2 days (*n* = 247)	2–7 days (*n* = 169)	Greater than 7 days (*n* = 126)	
	(*n* = 94)				
Maternal age ≥35 years, *n* (%)	34 (36.2)	72 (29.1)	58 (34.3)	40 (31.7)	0.554
Nulliparity, *n* (%)	30 (31.9)	108 (43.7)	59 (34.9)	45 (35.7)	0.118
Female sex, *n* (%)	34 (36.2)	116 (47.0)	77 (45.6)	59 (46.8)	0.316
Type of diabetes, *n* (%)					**0**.**006**
GDM	73 (77.7)^a,b^	223 (90.3)	151 (89.3)	115 (91.3)	
DIP	21 (22.3)^a,b^	24 (9.7)	18 (10.7)	11 (8.7)	
Ways of glycemic control, *n* (%)					**<0**.**001**^c^
Diet only	53 (56.4)^b^	149 (60.3)^b^	81 (47.9)	46 (36.5)	
Insulin and diet	35 (37.2)^b^	93 (37.7)^b^	81 (47.9)	78 (61.9)	
Medication and diet	6 (6.4)	5 (2.0)	7 (4.1)	2 (1.6)	
PPROM, *n* (%)	18 (19.1)^d^	83 (33.6)^b,d^	9 (5.3)	14 (11.1)	**<0**.**001**
Placenta previa, *n* (%)	11 (11.7)^b,d^	44 (17.8)^b,d^	58 (34.3)	48 (38.1)	**<0**.**001**
Cesarean delivery, *n* (%)	65 (69.1)^b,d^	195 (78.9)^b,d^	163 (96.4)	114 (90.5)	**<0**.**001**
Indication for delivery, *n* (%)					**<0**.**001**
Spontaneous^e^	39 (41.5)^b,d^	129 (52.2)^b,d^	24 (14.2)	25 (19.8)	** **
Fetal^f^	18 (19.1)	32 (13.0)	32 (18.9)	26 (20.6)	** **
Maternal^g^	37 (39.4)^b,d^	86 (34.8)^b,d^	113 (66.9)	75 (59.5)	** **
Hypertensive disorders, *n* (%)	33 (35.1)	60 (24.3)	45 (26.6)	30 (23.8)	0.198
BMI, mean ± SD	29.7 ± 6.6	29.4 ± 4.9	29.0 ± 4.4	28.5 ± 4.3	0.337
HbA1c in second or third trimester, %, median (range)	5.9 (5.1–14.0)^a,b,d^	5.7 (4.7–13.7)^b^	5.7 (4.3–10.4)^b^	5.6 (3.8–7.9)	**<0**.**001**
GA at ACS, median (range)	—	35.9 (33.7–36.9)	35.3 (33.3–36.6)	33.6 (26.0–35.6)	** **
GA at delivery, mean ± SD	35.8 ± 0.8	35.8 ± 0.8	35.7 ± 0.8	35.6 ± 0.8	0.356
Birth weight (g), mean ± SD	2,892.5 ± 677.1^b^	2,806.6 ± 564.5^b^	2,754.5 ± 582.1^b^	2,548.3 ± 509.6	**<0**.**001**
1-min Apgar score <7, *n* (%)	5 (5.3)	20 (8.1)	11 (6.5)	9 (7.1)	0.821
Large for gestational age, *n* (%)	22 (23.4)^b^	46 (18.6)	23 (13.6)	11 (8.7)	**0**.**013**
Small for gestational age, *n* (%)	9 (9.6)	18 (7.3)	17 (10.1)	19 (15.1)	0.128

Bold values are statistically significant. *Post hoc* method by Bonferroni.

^a^ Means vs. delivered <2 days after ACS administration group.

^b^ Means vs. delivered >7 days after ACS administration group.

^c^ Means Fisher's exact probability method.

^d^ Means vs. delivered 2–7 days after ACS administration group.

^e^ Spontaneous indications for delivery included preterm labor, preterm prelabor rupture of membranes, and cervical insufficiency.

^f^ Fetal indications for delivery included non-reassuring fetal status, intrauterine growth restriction, oligohydramnios or anhydramnios, and fetal anomaly.

^g^ Maternal indications for delivery included preeclampsia or gestational hypertension, abnormal vaginal bleeding (such as placenta previa and placental abruption), and other maternal medical conditions.

**Table 2:** Comparison of indications for antenatal corticosteroid administration among groups.

Variables	Antenatal corticosteroids			*P*
	Less than 2 days (*n* = 247)	2–7 days (*n* = 169)	Greater than 7 days (*n* = 126)	
Indications for ACS, *n* (%)				**<0**.**001**^a^
Preterm labor, *n* (%)	50 (20.2)	11 (24.6)	18 (14.3)	
PPROM, *n* (%)	74 (30)	4 (2.4)	2 (1.6)	
Fetal indications, *n* (%)	30 (12.1)	40 (23.7)	30 (23.8)	
Maternal indications, *n* (%)	87 (35.2)	106 (62.7)	59 (46.2)	
Abnormal vaginal bleeding, *n* (%)	6 (2.4)	7 (4.1)	16 (12.7)	
Asymptomatic changes of the cervix, *n* (%)	0 (0)	1 (0.6)	1 (0.8)	

Bold values are statistically significant.

^a^ Means Fisher's exact probability method.

**Table 3:** Neonatal outcomes according to antenatal corticosteroid administration to birth interval.

Outcome	No antenatal corticosteroids	Antenatal corticosteroids			*P*
		Less than 2 days (*n* = 247)	2–7 days (*n* = 169)	Greater than 7 days (*n* = 126)	
	(*n* = 94)				
Primary outcomes					
RDS (including TTN), *n* (%)	14 (14.9)	29 (11.7)	27 (16.0)	26 (20.6)	0.152
Hypoglycemia, *n* (%)	23 (24.5)	65 (26.3)	40 (23.7)	22 (17.5)	0.297
Secondary outcomes					
Resuscitation at birth, *n* (%)	8 (8.5)	18 (7.3)	13 (7.7)	11 (8.7)	0.959
Mechanical ventilation, *n* (%)	3 (3.2)	4 (1.6)	7 (4.1)	3 (2.4)	0.423^a^
CPAP or HFNC, *n* (%)	15 (16.0)	37 (15.0)	33 (19.5)	32 (25.4)	0.088
Oxygen requirement, *n* (%)	18 (19.1)	48 (19.4)	38 (22.5)	36 (28.6)	0.205
Need for surfactant, *n* (%)	4 (4.3)	5 (2.0)^b^	9 (5.3)	11 (8.7)	**0**.**026**^a^
The lowest glucose level, mean ± SD	2.9 ± 1.1	2.9 ± 1.0	2.9 ± 0.9	2.9 ± 0.7	0.887
Neonatal ward admission, *n* (%)	71 (75.5)	190 (76.9)	138 (81.7)	111 (88.1)	**0**.**043**
Days in neonatal ward, median (range)	9 (3–31)	9 (3–27)	10 (2–34)	11 (4–44)	0.197
Admitted—respiratory issue, *n* (%)	19 (20.2)	41 (16.6)	34 (20.1)	36 (28.6)	0.061
Admitted—hypoglycemia, *n* (%)	1 (1.1)	8 (3.2)	4 (2.4)	2 (1.6)	0.720^a^

Bold values are statistically significant. *Post hoc* method by Bonferroni.

^a^ Means Fisher's exact probability method.

^b^ Means vs. delivered >7 days after ACS administration group.

**Table 4:** Multivariable analysis of the association between the antenatal corticosteroid administration to birth interval and adverse neonatal outcomes.

Outcome	No antenatal corticosteroids	Antenatal corticosteroids [*P*, aOR (95% CI)]		
		Less than 2 days	2–7 days	Greater than 7 days
Primary outcomes				
RDS (including TTN)	Ref	0.496–0.749 (0.326–1.721)	0.817–0.901 (0.374–2.172)	0.665–1.226 (0.487–3.090)
Hypoglycemia	Ref	0.197–1.631 (0.775–3.432)	0.679–1.184 (0.532–2.633)	0.781–0.881 (0.362–2.148)
Secondary outcomes				
Resuscitation at birth	Ref	0.732–0.830 (0.287–2.404)	0.464–0.647 (0.202–2.076)	0.558–0.693 (0.203–2.369)
Mechanical ventilation	Ref	0.629–0.632 (0.099–4.055)	0.942–1.072 (0.166–6.938)	0.833–0.801 (0.102–6.287)
CPAP or HFNC	Ref	0.941–0.971 (0.439–2.147)	0.753–0.873 (0.375–2.032)	0.624–1.248 (0.515–3.022)
Oxygen requirement	Ref	0.999–1.000 (0.477–2.095)	0.859–0.931 (0.422–2.053)	0.622–1.233 (0.536–2.835)
Need for surfactant	Ref	0.300–0.443 (0.095–2.068)	0.902–0.914 (0.217–3.850)	0.818–1.191 (0.268–5.286)
Neonatal ward admission	Ref	0.666–1.193 (0.535–2.658)	0.576–1.287 (0.532–3.111)	0.316–1.649 (0.620–4.387)
Admitted—respiratory issue	Ref	0.476–0.768 (0.371–1.589)	0.690–0.853 (0.390–1.863)	0.461–1.357 (0.603–3.055)
Admitted—hypoglycemia	Ref	0.498–2.181 (0.299–20.081)	0.513–2.295 (0.191–27.591)	0.933–0.879 (0.403–17.840)

aOR, adjusted odds ratio; Ref, reference.

Adjusted for type of diabetes, ways of glycemic control, PPROM, placenta previa, cesarean delivery, indication for delivery, large for gestational age, birth weight, and HbA1c.

## Discussion

In our study, ACS treatment did not reduce the incidence of respiratory system diseases in LPT neonates born to diabetic mothers. In addition, it did not increase the incidence of neonatal hypoglycemia, regardless of the time interval to delivery.

The ALPS trial found that administering ACS significantly reduced the incidence of neonatal respiratory complications in LPT infants (8). However, the trial included only a small sample of women with GDM (306/2,831), and it excluded women with GDM requiring insulin treatment or those with pregestational diabetes. Dude et al. (18) and Krispin et al. (19) conducted retrospective cohort studies of diabetic mothers. One study included women with all types of diabetes, and ACS treatment was administered exclusively during the LPT period (18). The other study included 161 mothers with GDM who delivered during the LPT period and 2,101 mothers with GDM who delivered at term. The ACS group included women who received ACS treatment between 24 + 0 and 33 + 6 weeks of gestation (19). In contrast to these studies, our study included women with any type of diabetes and those treated with ACS at any time during pregnancy as a case group. Despite the differences, their findings are consistent with ours in that ACS treatment was not associated with reducing respiratory morbidity in LPT infants born to diabetic mothers. Furthermore, some studies found that ACS treatment does not improve neonatal respiratory outcomes even in mothers with diabetes who delivered between 23 + 0 and 33 + 6 weeks or underwent early-term scheduled cesarean section (ETSCS) (26–28). ACS treatment can result in transient maternal hyperglycemia and subsequent fetal hyperinsulinemia, which leads to delayed surfactant synthesis (29–31). In addition, Refuerzo et al. found that hyperglycemia following ACS treatment is more severe in diabetic pregnant women, with glucose levels increasing nearly 50% higher than that in non-diabetic pregnant women (32). Therefore, neonates born to diabetic women may not receive the same benefits from ACS, compared with those born to mothers without diabetes.

Studies have found that ACS may increase the incidence of neonatal hypoglycemia by inducing transient maternal hyperglycemia, leading to fetal reactive hyperinsulinemia and fetal adrenal suppression (33–35). In addition, previous experimental studies demonstrated that ACS exposure may induce the production of fetal hepatic enzymes involved in regulating glucose metabolism (36, 37). Thus, ACS exposure may further increase the risk of hypoglycemia in infants born to diabetic mothers. However, the effect of ACS on the blood glucose levels of neonates has been inconsistent across different studies. The ALPS trial, which included only a small sample of women with GDM (306/2,831), also showed that ACS could significantly increase the incidence of neonatal hypoglycemia [relative risk: 1.6; 95% confidence interval (CI): 1.37–1.87; *P* < 0.001] (8). The study of Dude et al. found a higher incidence of hypoglycemia in newborns born to mothers with diabetes treated with LPT corticosteroids (adjusted odds ratio 2.96, 95% CI: 1.29–6.82) (18). Two retrospective cohort studies by Gupta et al. (27) and Li et al. (28) on women with all types of diabetes recently found that ACS treatment before ETSCS was related to more neonatal hypoglycemia. Moreover, Li et al. found that this association only existed in newborns born within 2 days after ACS administration. However, our study found no association between ACS administration at any time during pregnancy and neonatal hypoglycemia in the population of pregnant women with diabetes. The study by Krispin et al. (19) and another study (38), which included 54 mothers with pregestational diabetes who delivered during the LPT period, also did not find a correlation between the two. Although none of the studies took into account the timing of administration, maternal hyperglycemia following corticosteroid treatment may be transient. The influences of ACS on maternal and subsequent fetal glucose homeostasis may disappear or decrease with the extension of the time interval between ACS administration and delivery (39). In the future, larger prospective studies are required to assess the effects of ACS exposure in this particular group.

In addition, in our study, the admission rate for hypoglycemia was much lower than the incidence of neonatal hypoglycemia. In China, apart from issues such as respiratory problems, jaundice, and infections, many neonates are hospitalized due to the lack of confidence of their parents in taking care of premature infants. As a result, many cases of neonatal hypoglycemia were detected during hospitalization, which is also one of the reasons for the high rates of hospitalization in our study. Therefore, regardless of hospitalization, routine blood glucose monitoring should be conducted for LPT infants born to mothers with diabetes.

### Strength and limitations

Our study has several strengths. To our knowledge, this is the first study to assess the association between different time intervals from ACS administration to delivery and neonatal outcomes in LPT infants born to diabetic mothers. Another strength of our study is the sample size, which is the largest among reports focusing on this particular population. Our study also has several limitations. As a retrospective cohort study, some confounding factors that might have influenced the results were not reported, such as blood glucose levels in the third trimester of pregnancy. However, we collected HbA1c levels in the third trimester as a measure. In addition, because our hospital is the largest tertiary medical center in Henan province, we have many high-risk parturients and a high rate of cesarean deliveries in the study population. Lastly, due to the high rate of ACS exposure in our hospital, there was a difference in sample size between our case groups and the control group, which may have influenced the results. Therefore, a larger randomized controlled trial is needed to confirm these findings in the future.

## Conclusions

In conclusion, our findings suggest that ACS treatment is not associated with neonatal hypoglycemia and respiratory outcomes of LPT neonates born to mothers with any type of diabetes, regardless of the time interval to delivery. Further studies are required to evaluate the effect of ACS treatment in this specific population.

## Data Availability

The original contributions presented in the study are included in the article/Supplementary Material, further inquiries can be directed to the corresponding author.
